# An Animal Model for Mammalian Attachment: Infant Titi Monkey (*Plecturocebus cupreus*) Attachment Behavior Is Associated With Their Social Behavior as Adults

**DOI:** 10.3389/fpsyg.2020.00025

**Published:** 2020-01-28

**Authors:** Logan E. Savidge, Karen L. Bales

**Affiliations:** ^1^Department of Psychology, University of California, Davis, Davis, CA, United States; ^2^Psychology Graduate Group, University of California, Davis, Davis, CA, United States; ^3^California National Primate Research Center, Davis, CA, United States

**Keywords:** attachment, pair bond, non-human primate, social bonding, novelty

## Abstract

Close social bonds are integral for good health and longevity in humans and non-human primates (NHPs), yet we have very little understanding of the neurobiological differences between healthy and unhealthy relationships. Our current understanding of social bonding is grounded in Bowlby’s theory of attachment. Work done with human infants and adult couples has suggested that attachment behavior developed in infancy remains stable through development into adulthood. Unfortunately, knowledge of the neurobiological correlates of attachment behavior has been limited due to a lack of animal models with both infant and adult attachments similar to humans. To address this, we measured behavioral responses to separation from their primary attachment figure in infant and adult titi monkeys (*Plecturocebus cupreus*). In Experiment 1, we tested for a linear relationship between the subject’s response to separation as an infant and their response to separation as an adult. We found greater decreases in infant locomotor behavior in the presence, as opposed to absence, of their primary attachment figure to be indicative of decreased anxiety-like behavior in the presence, as opposed to absence, of their adult pair mates during a novelty response task. In Experiment 2, we increased our sample size, accounted for adverse early experience, and tested a different outcome measure, adult affiliative behavior. We hypothesized that the level of intensity of an infant’s response to separation would explain affiliative behavior with their mate as an adult, but adverse early experience could change this relationship. When we compared infant response to separation to adult affiliative behavior during the first 6 months of their first adult pair bond, we observed a linear relationship for infants with typical early experience, but not for infants with adverse early experience. Infants with a greater change in locomotive behavior between the father and alone conditions were more affiliative with their first adult pair mate. These data support the use of titi monkeys as an appropriate animal model for further investigation of the neurobiology underlying attachment behavior.

## Introduction

Both humans and non-human primates (NHPs) rely on close social bonds to survive and thrive in their environments (Berkman and Syme, 1979; House et al., 1988; Holt-Lunstad et al., 2010; Stanton and Campbell, 2014). Consequently, expanding our knowledge of the underlying biology of social bonding is important for understanding the impact social bonds have on mental and physical health outcomes. For humans, common social bonds can take the form of friendships, familial bonds, or romantic partnerships. Infant-parent and adult romantic relationships are further characterized by forms of attachment as described in Attachment Theory (Bowlby, 1969; Hazan and Shaver, 1987). The current article is written from a developmental perspective, but it should be noted that Attachment Theory has been historically discussed within different psychological contexts (for a detailed review see George and West, 1999). For both infant-parent and adult romantic relationships, the attachment is defined by three distinct behaviors: proximity maintenance, distress upon involuntary separation, and the ability of the attachment figure to ameliorate stress during anxiety-provoking instances (Bowlby, 1969; Hazan and Shaver, 1987; French et al., 2018). While these three behaviors are the keystones of attachments and relationships, they vary between and within individuals, reflecting the quality of the bond. Infantile attachment behavior has been extensively studied in non-human animal models, but adult attachment, or pair bonding, is largely unexplored in animal models including NHPs. Developing an NHP model capable of illustrating individual variation in attachment behavior, similar to that in humans, from infancy to adulthood could open opportunities to understand the intricate effects of attachments on behavior, cognition, and biology.

The mother–infant bond has been similarly characterized in non-human animals and humans, with infants categorized as secure, insecure/anxious, or insecure/avoidant (Harlow and Zimmermann, 1959; Bowlby, 1969, Bowlby, 1982; Ainsworth, 1979; Bard and Nadler, 1983; Vaughn and Waters, 1990; Kondo-Ikemura and Waters, 1995; Warfield et al., 2011; Numan, 2015). Adult attachment styles follow similar categories as infant attachment styles and can be measured through self-reports but are usually measured by coding observed interactions between partners (Hazan and Shaver, 1987; Vaughn and Waters, 1990; Slade et al., 1999). Both secure infants and adults exhibit confidence that their caregiver or partner will be available and responsive when needed, as illustrated by robust positive effects of their caregiver or partner’s presence during stressful situations and faster physiological recovery from stressful situations (Ditzen et al., 2008; Meuwly et al., 2012). Insecure/anxious infants and partners desire frequent interaction or contact while simultaneously exhibiting emotional distance and a reluctance to express closeness with their caregiver or partner (Ainsworth and Bell, 1970; Waters, 1978; Ainsworth, 1979). Anxiously attached individuals experience greater distress and, at times, increased anger toward their parents or partners in stressful scenarios compared to their securely attached counterparts (Feeney and Kirkpatrick, 1996; Rholes et al., 1999). In adulthood, avoidant and anxious partners exhibit jealousy and emotional extremes (Hazan and Shaver, 1987; Levy and Davis, 1988). In this study, our goal was to develop an NHP model to investigate the physiological and neurobiological processes that underlie these individual differences.

The frequency of infant attachment styles is paralleled in the adult population suggesting that attachment style may remain consistent throughout life (Bartholomew and Shaver, 1998; Fraley, 2002). However, some studies have found inconsistencies between infant attachment behavior and adult attachment styles. Weinfield et al. (2000) noticed an abnormally high distribution of insecure attachment styles in adults from high risk backgrounds, defined by the mother’s age, income, and whether or not the pregnancy was planned, compared to estimates from middle class adults without such risk factors. They believe this disparity could be due to high rates of childhood maltreatment and maternal depression. This proposition has been supported by research in animal models of maternal abuse in which the offspring grow up to develop atypical social behavior (Maestripieri et al., 2005; Rincón-Cortés and Sullivan, 2016). Changes in attachment behavior also vary depending on the type of attachment an infant initially develops. Human infants classified as secure, who consequently experience stressful life events are more likely to become insecurely attached as adults than insecure infants experiencing typical experiences are to become securely attached (Waters and Merrick, 2000). Individuals with unstable attachment figures also exhibit more variability in attachment behavior compared to individuals who have stable relationships with their attachment (Jones et al., 2018).

Given the difficulty of tracking human attachment behavior through the lifespan across a multitude of different bonds form from infancy to adulthood (Fraley, 2019), we sought to explore attachment behavior in a New World monkey, the coppery titi monkey (*Plecturocebus cupreus*, formerly known as *Callicebus cupreus*). While a young adult human in their twenties may have already experienced a variety of intimate bonds, titi monkeys in the laboratory offer an opportunity to directly study the relationship between an infant’s attachment to their parent and the same subject’s attachment to their first mate. Infant titi monkeys form a specific attachment to their fathers, exhibiting distress upon separation, increased exploration in the father’s presence, and proximity maintenance (Hoffman et al., 1995; Spence-Aizenberg et al., 2016). Previous work with infant titi monkeys revealed effects of adverse early experience in the response of titi monkey infants to separation from the attachment figure and exposure to a novel environment. Larke et al. (2017) ran a modified open field test in which infant titi monkeys were placed in an open field with the opportunity to move about the new environment freely and engage with a novel object. The infants were then either left alone in the open field or allowed to interact with their mother, father, or sibling through a mesh grate (Larke et al., 2017). Infant titi monkeys with adverse early experience were less likely to maintain proximity to their father and exhibited more exploratory behavior during the separation condition (Larke et al., 2017). This work was the first to show variability in attachment behavior of infant titi monkeys. The current study aims to expand on these findings by examining consistencies in attachment behavior between the father–infant bond and the adult pair bond.

Adult titi monkeys form pair bonds that are similar to those observed in humans and can be summarized by the following behaviors and responses: proximity seeking (contact, preference, and exclusivity), separation distress (increased vocalization rate, heart rate, cortisol, and locomotion), and stress buffering (reduced vocalization rate, heart rate, and cortisol) (Mason and Mendoza, 1998). Behavioral and neurological variation can be observed within the first 48 h of pairing, shifting toward more affiliative behaviors and altered neural activity in the nucleus accumbens and ventral pallidum (Bales et al., 2007). There are also differences in behavioral maintenance of the pair bond, depending on individual temperament. For example, individual variation in aggression has been shown to predict affiliation within a pair. More characteristically aggressive males (which show higher levels of mate-guarding or “jealous” behavior) tended to be less affiliative with their partners (Witczak et al., 2018).

## Current Study

### Experiment 1

The current study examined the relationship between infant attachment behavior and adult attachment behavior in the titi monkey. We collected data on attachment behavior through a variety of measures. Subjects were tested in the presence and absence of their father and mother (as infants), or pair mate (as adults). Based on the Ainsworth Strange Situation paradigm we used a novel situation to provoke a psychological threat to activate attachment systems, measuring the subject’s behavior during a father, mother, and an alone condition (Ainsworth and Bell, 1970). We used the modified infant open field (IOF) test from Larke et al. (2017) to examine infant behavior during a novel experience in the presence and absence of their father. We were unable to use an open field task in adults, because they would be able to jump out of the arena. Therefore, adult behavior was assessed with a different novelty response task designed based on previous research showing anxiety-like behavior in response to novelty (Hennessy et al., 1995). During the task, the animals are trained to approach a wire box containing a series of unfamiliar patterns, which range from a blank sheet to complex patterns, and retrieve a piece of banana. The task reliably elicits behavioral inhibition in response to novel patterns (Arias del Razo et al., 2019). Although our infant task differs from our adult task, each paradigm achieves the overarching goal of activating the attachment system through exposure to anxiety-provoking situations (Bowlby, 1982; Simpson et al., 1992).

Given that infant titi monkeys form a primary attachment to their father, we hypothesized that variation in titi infant response to the presence of their father, but not their mother, in the IOF test would be demonstrative of their attachment behavior as adults. Specifically, infants that responded to the sight of their father during the IOF test with increased contact calls, decreased locomotion, and increased time spent at the grate, which are examples of the infant seeking proximity and comfort during a stressful situation, would also receive the most benefit from their partner’s presence during the novelty response task as adults (Hoffman et al., 1995). In adulthood, we expected to see similar individual variation in behavioral responses to involuntary separation from their pair mate as we had observed in the IOF test. We hypothesized that the reaction to involuntary separation during the alone condition would inhibit behavioral response during testing. We also hypothesized that individuals that were more affiliative with their pair mate would have a stronger reaction to separation from their pair mate and would therefore be less likely to participate in the task than subjects that showed less affiliation with their pair mate.

### Experiment 2

Following Experiment 1, we investigated the relationship between infant behavior, life experience, and adult pair behavior more specifically. We began with the same infant data from subjects’ 4-month IOF test but this time we coded any/all adverse experiences the subject experienced during development. We took special note of adverse experiences occurring after the subject’s IOF test that may have changed their attachment behavior, but considered all adverse experience when examining group differences because we cannot be certain of when or how these experiences will affect behavior (Opendak and Sullivan, 2016). These data were then entered into a linear model predicting affiliative behavior in the subjects’ first adult pair bond. We hypothesized that infants exhibiting strong attachments to their fathers, evidenced by an increased behavioral response to his absence, would also exhibit more affiliative behavior in their first adult pair bond. We also expected to see an effect of adverse experience on this relationship such that behavior in the IOF test would not be adequate explanation of variance in adult affiliative behavior if the infants experienced adversity during development.

## Materials and Methods

### Subjects

#### Experiment 1

Subjects were 11 captive-born titi monkeys (*P. cupreus*), five males and six females, housed at the California National Primate Research Center (CNPRC) in Davis, California. All subjects were tested at two time points: 4 months of age and adulthood between 27 and 118 months old (mean age = 51.6 months, SD = 34.7 months). Infants were housed in their natal group and once subjects reached adulthood, they were removed from their natal group and housed with an unfamiliar opposite sex pair mate in 1.2 m × 1.2 m × 1.8 m cages. Pairs were determined by the experimenters based on lack of genetic relatedness, to avoid inbreeding in the colony. All animals were housed indoors and fed twice daily at 09:00 h and 13:00 h with water available *ad libitum*. Their diet consisted of a commercial primate chow diet supplemented with rice cereal, carrots, bananas, apples, and raisins. Husbandry training and caging were the same as previously described in Valeggia et al. (1999) and Tardif et al. (2006).

#### Experiment 2

Subjects were 25 captive-born titi monkeys (*P. cupreus*), 12 males, and 13 females, housed at the CNPRC. All subjects were tested in the IOF test at 4 months of age. Of the 25 subjects, 11 were from Experiment 1. As adults, they were observed every 2 h from 08:30–16:30 h for 6 months following their first pairing (mean age = 26.2 months, SD = 9 months). Subjects were housed and fed identically to Experiment 1. All procedures were approved by the University of California, Davis Institutional Animal Care and Use Committee.

### Experimental Design

#### Infant Open Field

The testing apparatus was made to resemble an open field similar to those used in rodent studies (Gould et al., 2009). The paradigm was adapted for infant titi monkeys with walls constructed 1 m high around a base 1 m wide by 1 m long. Walls were made out of opaque white polyvinyl chloride to limit visibility to the surrounding area. As in rodent open field tests, the floor was marked with gridlines to indicate specific locations within the field. A wire mesh grate was built into one of the walls to allow visual, auditory, and olfactory access to the infant’s father or mother. At the start of testing, a small piece of brown felt was placed on the left side of the open field (with respect to the wire grate) to serve as a novel object. A familiar food reward, most often a peanut, was placed on the right side of the open field. The field was illuminated by bright overhead lights.

Testing was conducted between 06:00 and 08:00 h. Subjects and their family members were caught in transport boxes (0.3 m × 0.3 m × 0.6 m in size) made of white opaque plastic and wire mesh. Adults and older siblings were caught in individual boxes while the subject would share a box with one of their family members, most often their father. The transport boxes were then covered with a towel and brought to a separate room to eliminate auditory and olfactory stimuli from other monkeys.

The full test consisted of four randomized trials in which an empty transport box, a transport box with the mother, the transport box with the father, or the transport box with a sibling were placed in front of the grate. If the subject did not have a sibling, they were exposed to the empty transport box for an additional trial. The current study did not analyze infant behavior during the sibling condition, and if the infant did not have a sibling we analyzed the first of the two possible empty conditions to avoid exacerbation of the stress response due to extra time alone in the open field (Larke et al., 2017).

#### Novelty Response Task

This study employed a within-subjects design with “social” and “alone” conditions counterbalanced. There was a minimum of 3 weeks between testing conditions for all subjects. Both testing sessions were a minimum of 6 months after the subject had been paired. Six months was selected based on previous experiments that show titi monkeys have a consistent behavioral preference for the new pair mate after 6 months of pairing (Rothwell et al., submitted). Average pair tenure for the current subjects was 18.2 months (*SD* = 9 months). The novelty response task was used to assess the ability of a pair-mate’s presence to buffer an individual’s stress response. Previous work with titi monkeys shows that they are more inhibited and exhibit greater elevations in stress hormones in response to novelty than another, non-monogamous, New World monkey species, the squirrel monkey (*Saimiri sciureus*) (Hennessy et al., 1995). This study also showed that small incremental changes in novelty were enough to evoke an elevated cortisol response in the titi monkey.

For the task, we used a small wire box, hereafter referred to as the test box. The test box contained a card displaying the visual stimulus and a small ledge where a piece of banana reward could be placed. For the animal to have completed the task he/she must have approached the test box and reached toward the visual stimulus to retrieve the reward. Animals were first habituated to the test using a blank card in the testing box. Habituation could consist of up to 15 sessions with 10 trials in each session; however, none of the current subjects needed the maximum number of sessions. Subjects were habituated under both conditions, either alone or social before they were tested in the respective paradigm. To be considered habituated, the animal had to approach the test box and reach for the reward under 30 s for 10 consecutive trials. Once the animal met habituation criteria they began testing.

A single test consists of six trials in which the subject must complete the novelty response task. The difficulty of the task differed depending on which visual stimulus was presented. The six trials consisted of six cards from set of cards: a baseline card, four patterned cards ascending in complexity, and a final baseline card (Table 1). For the first and sixth “baseline” trials, the animal was shown a blank white card. During trials 2–5 the animal was shown increasingly complex patterns. An animal’s participation on the task was measured by the time it took for the animal to retrieve the reward on each independent trial. The animal was given 30 s to complete the task. Failure to retrieve the reward within that time frame was marked as a “balk” and interpreted as a refusal to participate. All patterns were black and white to control for sex differences in titi monkey color vision (Bunce et al., 2011).

**TABLE 1:** Descriptions of visual stimuli presented to the subjects during the novelty response task.

Trial number	Description of card content
Trial 1	Baseline-blank white background
Trial 2	A single line
Trial 3	Three different simple geometric shapes
Trial 4	Two different simple geometric shapes and two drawings of flowers
Trial 5	Eight elements against a background shaded differently than trials 2–4: two intersecting lines, two simple geometric shapes, two slightly more complex geometric shapes, and two drawings of flowers.
Trial 6	Baseline-blank white background

Each card that the animal was exposed to during testing was novel to that individual on the first day of testing. Testing in each paradigm was conducted across 4 days. If, for some reason, testing could not be completed consecutively, we made sure all four test days occurred within the same week.

For example:

Testing day 1: Card set #1 – six trials
Testing day 2: Card set #1 – six trials
Testing day 3: Card set #2 – six trials
Testing day 4: Card set #2 – six trials.

A total of six card sets were used for the experiment. Each individual was only tested on four of the six sets to ensure there were enough sets for their pair mate to be tested with a novel set during their social condition instead of reusing one that they may have seen by chance when their pair mate was being tested.

Testing was completed in the animal’s home cage either in the presence or absence of their pair mate. At the start of each test, the experimenter would enter the cage with a small familiar transport box. If it was the social condition, the experimenter would simply enter and exit the cage with the box in hand. In the alone condition, the experimenter would catch the pair mate in the transport box and take them out of the cage. The pair mate would wait out of sight of the subject. However, the cage mate remained within olfactory and auditory access of the subject and any vocalizations were audible to their pair mate. Once the cage mate had been removed, the test box was clipped to the side of the cage, and the test began. The subject was ushered to the back of the cage while the experimenter placed the card and the banana behind a visual barrier. A trial began with a count down, “3, 2, 1, start”, then the experimenter removed the barrier and exposed the designated card. The trial ended when the subject was observed reaching for the banana. If the subject did not reach for the banana within the 30-s time limit, the experimenter covered the card, counted that trial as a “balk,” and moved on to the next trial.

#### Zone Training

**FIGURE 1:**
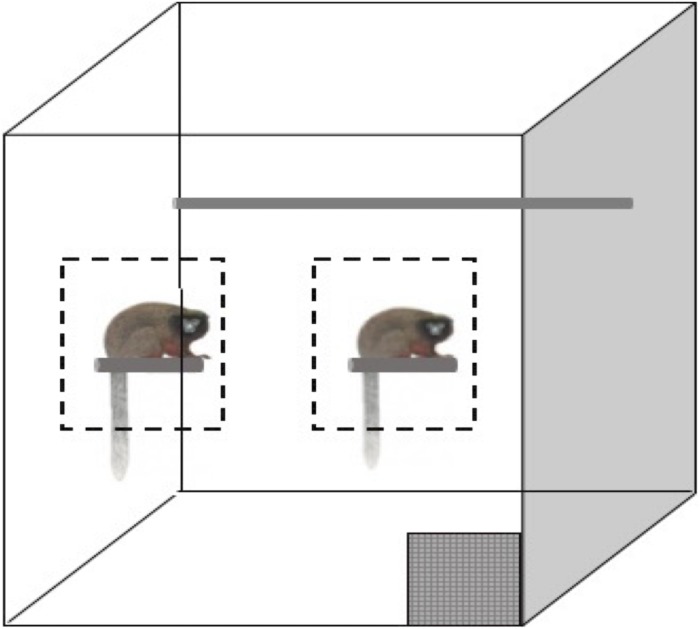
Simplified representation of the titi monkey home cage. The gray checkered box is the release door, the gray cylinder each animal is sitting on represents a perch and trained/testing zones are indicated by dashed outlines. Not to scale.

For the social condition, the subject and cage mates (some subjects had offspring in the cage) were trained to approach and remain in a designated zone as not to interfere with each other’s testing. During training, two experimenters would stand outside the cage and call the animals forward to specific zones. The zones were initially determined based on the apparent preference of each animal. The animal could choose to approach a small perch on the left side of the cage, known to staff as the enrichment perch where they were frequently given enriching foods (grains, rice cereal, and greens), or the animal could approach the right side of the cage where their food bowl was mounted (Figure 1).

Once the subject chose a zone, the experimenter would begin positive reinforcement training with a clicker. The goal was to reward the animal each time they observed their cage mate receive a treat while waiting in their own zone. During each session, both animals were trained to participate but only the test subject received the clicker training to avoid confusion with extra click sounds. Once animals showed a readiness to approach their zone and they were willing to remain in the zone for an entire 5-min session, the subject moved on to habituation for the novelty response task.

#### Affiliation Data

Scan sampling data on affiliation were used to determine baseline levels of affiliation between pairs in this study. These data were collected through cage-side checks, which were performed every 2 h 5 d a week for 6 months before each testing condition. Animals were scored for the following behaviors: contact (any bodily contact between the pair mates), proximity (within one arm’s reach of their pair mate), and tail-twining (pair mates sitting side-by-side with their tails wrapped together). If the animals were not engaging in any of these behaviors, they were marked as “none” for that observation. The mean number of checks per day for all subjects was 4.98 with a standard deviation of 1.02.

Values were then calculated for each subject from the pair check data for 6 months prior to testing in Experiment 1 or 6 months after pairing for Experiment 2. The values are the mean ratio calculated by dividing the number of observations the pair was observed in contact or proximity by the total number of observations that day. One of our subjects did not have a tail, due to necessary medical intervention, so we decided not to compare tail twining behavior for any of our subjects. Contact and proximity values (Exp. 1 *M* = 0.36, *SD* = 0.08; Exp. 2 *M* = 0.21, *SD* = 0.09), calculated from an average of 558 observations, indicate the percentage of scan samples participants were observed in contact or proximity each day, respectively.

#### Adverse Early Experience Classification

Infant experience was classified as adverse similar to Larke et al. (2017). We classified infant experience as adverse if the infant experienced a loss of a parent, a traumatic injury, or a significant separation from their attachment figure sometime before 9 months of age. We chose 9 months because that is the typical age our laboratory observes the infant behaving completely independent; behaviorally, the infant is no longer nursing or clinging to a parent.

### Data Analysis

#### Infant Open Field

For IOF behavior we analyzed all 25 subjects from Experiments 1 and 2 together. All locomotion, grate touch, and grate zone data were scored using Behavior Tracker 1.5^1^. The current study used the same ethogram as Larke et al. (2017) for measures of locomotor behavior (i.e., gridline cross) and proximity seeking behavior (i.e., grate touch and grate zone positioning). High levels of locomotor behavior are interpreted as increased arousal and high levels of proximity seeking behavior are indicative of the infant attempting to approach the stimulus on the other side of the grate. According to the Shapiro–Wilk normality test frequency of gridline crosses was not normally distributed in our sample (*W* = 0.78, *p* < 0.001). We scored the number of vocalizations each subject emitted during testing RavenLite2.0 (Bioacoustics Research Program 2014, Ithaca, NY, United States) software. Vocalization frequency data were normally distributed (*W* = 0.98, *p* = 0.23) with high levels of vocalization indicating increased arousal and proximity seeking behavior.

To account for non-normal data, we chose to run a linear mixed model (LMM) based on its robustness to abnormal distributions (Arnau et al., 2012) in R Statistical Software (version 3.2.2, R Core Team, 2018). Considering infant titi monkeys’ primary attachment to their father, we did not initially include infant behavior from the mother condition in our analyses. Our full model examined changes in behavior from the empty condition to the father condition and whether sex or the order of the condition in which the subject was exposed to first altered their behavior (fixed effects) with subject ID and day of testing as a random effect to account for repeated measures. After running our model, it became clear that there were no significant interactions or effects of sex, order of test condition, or day on behavior; we therefore elected to collapse the data set so that there was only one value per subject per behavior per condition (the mean value across all test days). The condensed data were normally distributed (*W* = 0.95, *p* = 0.19) and we performed one-way ANOVA’s with Tukey’s honestly significant difference (HSD) *post hoc* compare behavior between conditions.

#### Participation in the Novelty Response Task by Trial

All data were analyzed using R Studio (R Core Team, 2018). A Shapiro–Wilk test for normality revealed a heavy right skew in the latency data (Royston, 1982). The skew in the data was due to a right censorship of data where subjects balked. To account for the skew in the data we transformed the data into a binomial distribution indicating whether or not a subject participated in the given amount of time. To examine the effects of trial on participation behavior we ran a generalized LMM with trial condition as fixed effects and subject ID as a random intercept. Using emmeans package in R studio (version 3.2.2, R Core Team, 2018) we compared the estimated marginal means of each trial to determine which were statistically significant. These *post hoc* comparisons were done with pairwise, two-tailed, *t*-tests.

#### Experiment 1

To examine the effects of condition, affiliation with pair mate (measured by observed contact and proximity), order of condition, and sex on percent of participation in: all trials combined, easy trials (levels 1, 2, 3, and 6), and hard trials (levels 4 and 5), we calculated an average percentage of participation across all 4 days of testing to transform the data to continuous variables for a LMM. Trial types were identified as easy or hard by previous models comparing subject participation in each trial. Full models included the fixed effects: testing condition, order of condition, sex, pair experience (whether or not this was the subject’s first pair mate), order of conditions by type of condition interaction, sex by type of condition interaction, pair experience by condition interaction, and an order by condition interaction. To account for repeated measures all models included subject ID as a random intercept. We used a combination of backward selection and a loglikelihood ratio test combined with comparisons of Akaike information criterion (AIC) values to ensure we had the most parsimonious model (Supplementary Tables S1A,B). None of our independent variables explained more variance in participation during easy trials than the null hypothesis so they are not reported.

To remain consistent with our *a priori* model we included condition and affiliation scores in our final model regardless of whether they explained a significant amount variance. We constructed our *a priori* model from previous knowledge of affiliation and separation behavior in socially bonded species and hypothesized that the degree to which subjects express affiliation or respond to separation would relate to their behavioral response to separation from their pair mate (Ditzen et al., 2008).

For participation by infant behavior during father and empty conditions, data were analyzed similarly to our analysis of test condition with the addition of infant behaviors from our 11 subjects as independent variables. Based on *a priori* hypotheses, we only analyzed participation during the most difficult trials of the task which we believed most likely to activate the attachment system. Our full model contained infant locomotor, vocalization, grate zone, and grate touch behaviors as well as test condition and sex as fixed effects. We also included interaction terms between all infant behavior and test condition in anticipation that infant behavior would differentially explain adult participation depending on the condition. We then systematically removed insignificant effects through backward selection until we had the most parsimonious model with the lowest AIC value (Supplementary Table S1C). For participation by infant behavior during mother and empty conditions, we ran the same models as with the father condition with the exception of vocalization behavior (Supplementary Table S1D).

#### Experiment 2

To examine whether variability in adult affiliation could be explained by adverse early experience, we ran a linear regression with adverse early experience (yes or no) as the independent variable and adult affiliative behavior as the dependent variable. Adult affiliative behavior was defined as either the proportion of time the pair was observed in contact, or a combination of contact and proximity over the first 6 months of their first adult pair bond.

To test a possible relationship between infant behavior and adult affiliation we utilized linear regression starting with a full model containing all four measured infant behaviors (locomotion, vocalizations, grate touch behavior, and grate zone behavior) along with their interactions with IOF testing condition. We ran Shapiro–Wilk tests to confirm all four independent variables were normally distributed. We then selected the model with the smallest residual sum of squares for further interpretation.

## Results

### Infant Open Field

There were significant effects of condition on all observed behaviors regardless of which order the trials were presented. We did not observe any sex differences in infant behavior. Order and sex were therefore removed from the model due to non-significance. We elected to collapse the data set so that there was only one value per subject per behavior per condition (the mean value across all test days), and we performed independent sample’s *t*-tests to compare behavior between conditions.

**FIGURE 2:**
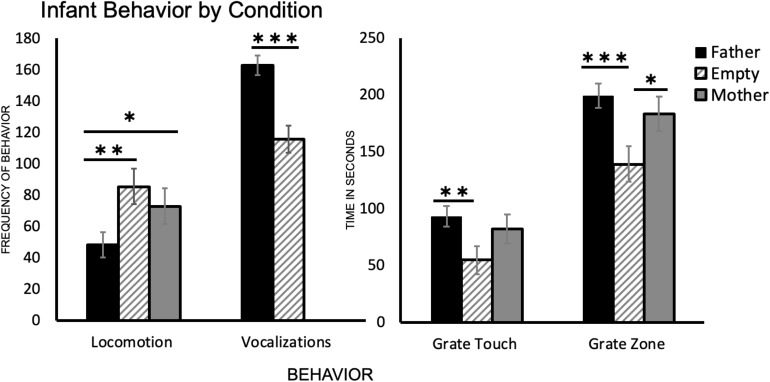
A summary of each infant behavior (*N* = 25) by condition. Locomotion represents the number of times an infant crossed a gridline, vocalizations are a count of all infant vocalizations during the trial, and grate touch plus grate zone behaviors are measured in seconds (total time per trial was 300 s). ^∗^*p* < 0.05, ^∗∗^*p* < 0.01, ^∗∗∗^*p* < 0.001.

One-way ANOVA followed by Tukey’s HSD *post hoc* comparisons revealed a significant difference between infant behavior in the father condition and the empty condition for all four behaviors. Subjects crossed more gridlines in the empty condition than in the father condition [*t*(39) = 3.26, *p* = 0.001], vocalized less in the empty condition than in the father condition [*t*(48) = −5.46, *p* < 0.001], spent less time touching the grate and in the grate zone in the empty condition than in the father condition [*t*(43) = −3.10, *p* = 0.002 and *t*(48) = −3.87, *p* < 0.001, respectively] (Table 2 and Figure 2).

**TABLE 2:** Mean and standard errors for our 25 subjects’ behavior across all 3 days of IOF testing.

Behavior	Condition	Mean	SE
Locomotor behavior	Empty	85.5	11.35
(gridline crosses)	Father	48.5	8.03
	Mother	72.8	11.39
Vocalizations	Empty	115.54	8.65
	Father	162.8	6.12
Grate touch	Empty	54.7	12.37
(in seconds)	Father	93.1	8.75
	Mother	82	12.42
Grate zone	Empty	139	15.35
(in seconds)	Father	199	10.86
	Mother	183	15.41

### Novelty Response Task

**FIGURE 3:**
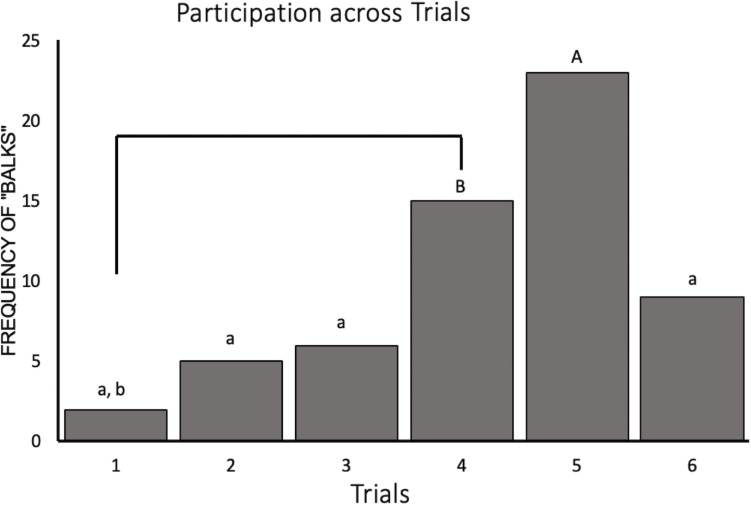
Novelty response task participation (*N* = 11) represented by the frequency of “balks” (i.e., instances where the animal refused to participate) plotted by trial. Trials labeled with a lower case “a” are significantly different from trial 5 (“A”) and trials labeled with a lower case “b” are significantly different from trial 4 (“B”).

Our generalized LMM containing trial and condition as fixed effects with subject ID as a random intercept significantly outperformed the null (χ^2^ = 41.17, *p* < 0.001). This model revealed a significant decrease in participation for trials 4 and 5 (*Z*_528_ = −3.06, *p* = 0.03 and *Z*_528_ = −3.97, *p* = 0.001, respectively) compared to participation for trial 1. Participation was also significantly lower in trial 5 compared to trials 2, 3, and 6 (*Z*_528_ = −3.75, *p* = 0.002, *Z*_528_ = −3.57, *p* = 0.005, and *Z*_528_ = −2.95, *p* = 0.04, respectively). The raw number of balks, instances where the monkey did not perform the task, by trial can be seen in Figure 3. We also saw an effect of social condition on task participation such that subjects were 1.9 times more likely to participate during the social condition than when they were alone (*Z*_528_ = 2.01, *p* = 0.05).

### Experiment 1

#### Participation in Novelty Response Task Trials by Adult Affiliation

##### Participation across all six trials

According to AIC scores and log likelihood comparisons, our final and most parsimonious model to explain novelty response task participation by condition and affiliation for all six trials combined and easy trials only included condition, affiliation (both contact and proximity measures), pair experience, and four interaction terms (condition by pair experience, condition by order, condition by contact, and condition by proximity) as fixed effects with subject ID as a random intercept to predict task participation (Supplementary Table S1A). With the current data we were unable to explain the variability in overall novelty response task participation with our hypothesized variables.

##### Participation during easy trials

Similarly, none of our models examining variance in participation during easy trials of the task outperformed the null (Supplementary Table S1B). Subject participation during easy trials does not appear dependent on testing condition or pair affiliation.

##### Participation during difficult trials

**FIGURE 4:**
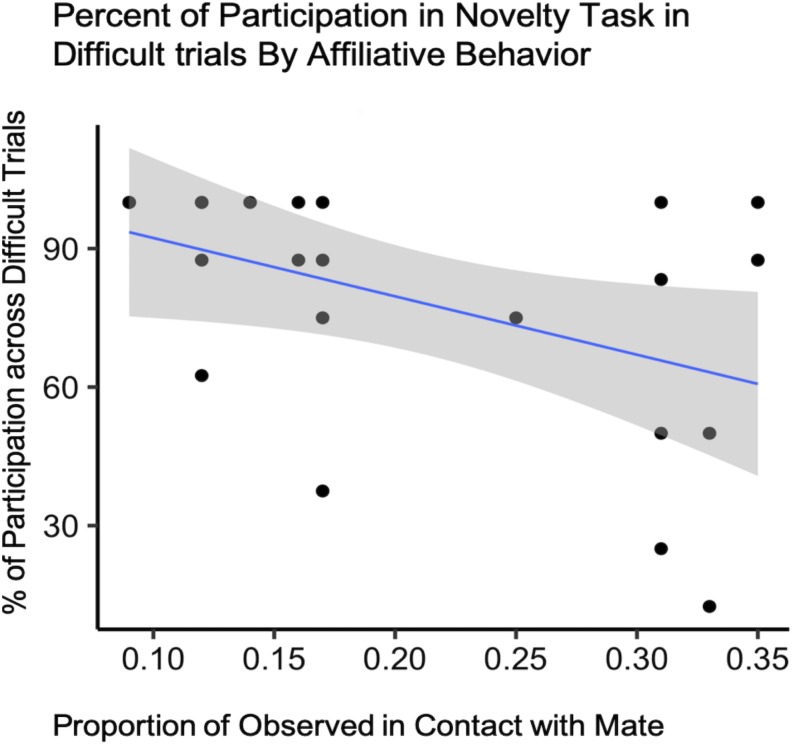
Novelty response task participation (*N* = 11) by Affiliative Contact. Percentage of subject participation across difficult trials plotted against the proportion of time subjects were observed in contact with their mate.

For difficult trials only, our final model included condition, affiliation (both contact and proximity measures), pair experience, and two interaction terms (condition by pair experience and condition by proximity) as fixed effects with subject ID as a random intercept to predict task participation (Supplementary Table S1C). Due to the small sample size, the expected variability from sample to sample was such that we cannot say that test condition explained more of the variance in our data than the null model [*b* = −19.00, CI 95% = (−74.672, 38.015)], (*t*_88_ = −0.70, *p* = 0.51) (Table 3). However, we did observe an effect of contact on participation with pairs observed in contact most often participating less in the novelty response task overall [*b* = −244.36, CI 95% = (−481.162, 12.183)], (*t*_88_ = −2.18, *p* = 0.048) (Table 4 and Figure 4).

**TABLE 3:** Summary of results for Experiment 1.

Predictor	Outcome	β	SE	*p*
*Adult affiliative behavior*	*Novelty response task participation during difficult trials*			
Social condition		−19.00	27.34	0.51
Pair experience		30.36	16.35	0.09
**Proportion of contact with mate**		**−244.36**	**112.22**	**0.05**
Proportion of proximity with mate		88.25	96.27	0.38
Social condition ^∗^ pair experience		**−**24.55	18.94	0.23
Social condition ^∗^ proportion of contact with mate		**−**26.16	125.15	0.84
Social condition ^∗^ proportion of proximity with mate		145.03	110.77	0.23

*Multiple linear regression model (*N* = 11) characterizing adult participation in the novelty response task from adult affiliative behavior. Boldface indicates statistically significant predictors.*

**TABLE 4:** Summary of Experiment 1.

Predictor	Outcome	β	SE	*p*
*Infant behavior during father condition with empty condition as the reference*	*Novelty response task participation during difficult trials*			
Adult social condition		35.16	52.20	0.52
Infant grate touch behavior (Dad)		−0.41	0.25	0.13
Infant grate zone behavior (Dad)		0.41	0.22	0.09
Infant vocalizations (Dad)		−0.15	0.17	0.40
Infant locomotor behavior (Dad)		−0.22	0.11	0.08
Social condition ^∗^ infant grate touch behavior		0.34	0.25	0.22
Social condition ^∗^ infant grate zone behavior		−0.52	0.24	0.08
Social condition ^∗^ infant vocalizations		0.38	0.31	0.25
**Social condition ^∗^ locomotor behavior**		−**0.52**	**0.24**	**0.03**

*Multiple linear regression model (*N* = 11) characterizing adult participation in the novelty response task from behavior in the father infant open field conditions. Boldface indicates statistically significant predictors.*

#### Adult Task Participation by Infant Behavior With Dad

**FIGURE 5:**
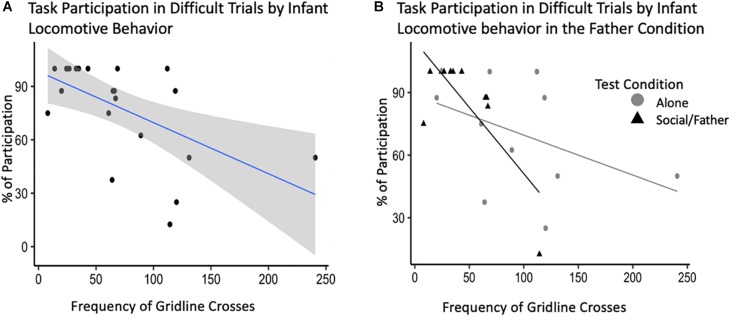
Novelty response task participation (*N* = 11) by infant behavior, specifically, locomotion. **(A)** Percent of task participation during difficult novelty response task trials plotted by infant locomotor behavior demonstrated by the average frequency of gridline crosses in a trial. **(B)** Interaction plot for the effects of test condition on the relationship between infant behavior and task participation.

After backward selection and AIC model comparisons, our final model explaining the relationship between adult task participation and infant behavior consisted of: infant locomotor behavior, vocalizations, grate touch and grate zone behavior, test condition, and the interactions between test condition and all infant behaviors as fixed effects, with subject ID as a random intercept (Supplementary Table S1D). Infant sex and vocalizations did not explain a significant portion of variability in adult participation in the difficult trials of the novelty response task. Our model showed an interaction between infant locomotor behavior and condition such that infant locomotion during the father condition explained a significant amount of the variance in participation during difficult trials of the novelty response task when their partner was present [*b* = −0.64, CI 95% = (1.37, −0.01)], (*t*_528_ = −2.60, *p* = 0.03) (Table 4). Infants that locomoted less when their father was present were more likely to participate in the difficult trials of the novelty response task when their partner was present as adults (Figure 5). We identified one potential outlier in our dataset and ran the same model without this value to investigate its effect on our observed associations. After excluding the data point with the highest frequency of gridline crosses, we were unable to explain variability in adult task participation. However, there was no theoretical reason to exclude this data point, so our following analyses will refer to the model including all observations.

#### Adult Task Participation by Infant Behavior With Mom

**FIGURE 6:**
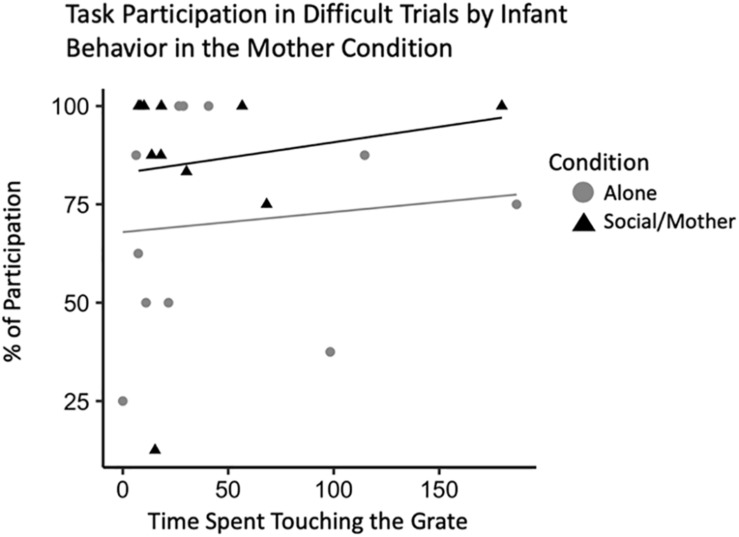
Novelty response task participation (*N* = 11) by infant behavior, specifically, time spent touching the grate. Interaction plot for the effects of test condition on the relationship between infant grate touching behavior and adult task participation during difficult novelty response task trials.

To confirm that this effect was related specifically to attachment behavior rather than general temperament, we ran the same model with infant locomotor behavior when their mother was present instead of their father. This model did not outperform the null suggesting infant behavior when their mother is present does not account for variability in adult behavior [*b* = 39.31, CI 95% = (−0.61, 87.04)], (*t*_528_ = 2.08, *p* = 0.07). There were also no interactions between locomotor behavior and condition [*b* = −0.24, CI 95% = (−0.45, −0.01)], (*t*_528_ = −2.09, *p* = 0.08). However, our model with infant behavior during the mother condition as an explanatory variable of adult participation in the novel pattern task did reveal some interesting effects. In the current sample, infants that tended to spend more time spent in the grate zone when their mother was present participated more in the difficult trials of the novelty response task [*b* = 0.44, CI 95% = (0.04, 0.85)], (*t*_528_ = 2.44, *p* = 0.04) (Table 5). There was also an interaction between grate touch behavior and adult participation indicating that infants that spent less time touching the grate when their mom was present participated more in the adult novel pattern task [*b* = 0.57, CI 95% = (0.09, 0.97)], (*t*_528_ = 2.82, *p* = 0.03) (Figure 6).

**TABLE 5:** Summary of results for Experiment 1 in mother condition.

Predictor	Outcome	β	SE	*p*
*Infant behavior during mother condition with empty condition as the reference*	*Novelty response task participation*			
Social condition		39.31	18.92	0.07
Grate touch behavior (mom)		−0.42	0.23	0.09
**Grate zone behavior (mom)**		**0.44**	**0.18**	**0.04**
**Locomotor behavior (mom)**		**−0.25**	**0.12**	**0.05**
**Social condition ^∗^ grate touch behavior**		**0.57**	**0.20**	**0.03**
Social condition ^∗^ grate zone behavior		−0.20	0.26	0.47
Social condition ^∗^ locomotor behavior		−0.24	0.11	0.08

*Multiple linear regression model (*N* = 11) of adult participation in the novelty response task from behavior in the mother infant open field condition. Boldface indicates statistically significant predictors.*

### Experiment 2

**FIGURE 7:**
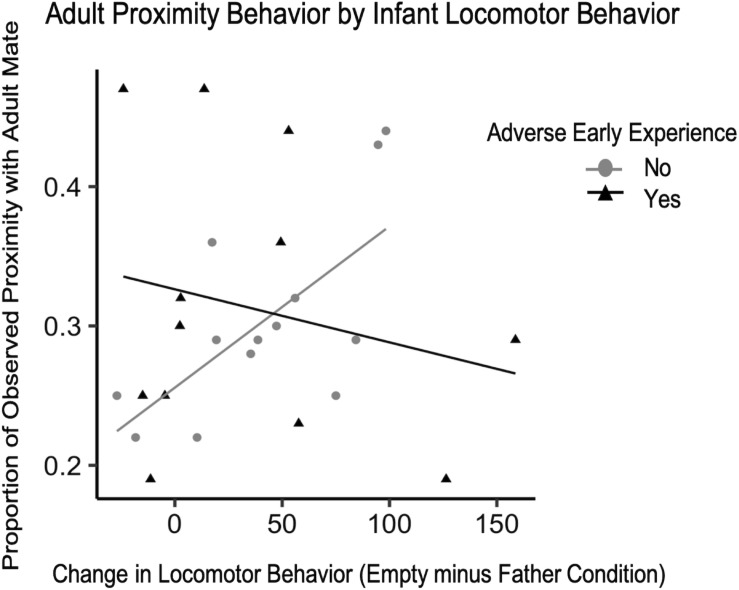
Difference scores for infant locomotor behavior (*N* = 25) plotted against the proportion of observations they were observed in proximity with their mate as adults with lines of best fit.

There were not enough instances of adverse experience that occurred before the 4-month IOF test to examine possible changes in behavior during the test, but previous work in our lab has shown a decrease in the time infants with adverse experience spend in the grate zone during the father condition compared to typically reared infants (Larke et al., 2017). Similar to Experiment 1, infant vocalization, grate touch, and grate zone behavior did not explain significant amounts of variability in adult affiliation and were therefore excluded from the final model. With the current data collected on adverse early experience, the difference in locomotor behavior from the father condition to the empty condition, and the interaction between these two independent variables and proportion of observed time spent in proximity or contact with their adult pair mate, we attempted to fit a model of linear growth and observe whether or not this accurately characterized the data compared to the null model. While our model had a lower residual sum of squares than the null, it did not significantly outperform the null model so we report the following findings with caution. Although group comparisons did not reveal a significant difference in adult affiliation by early experience for either contact or proximity behavior (*F*_16_ = 0.01, *p* = 0.92; *F*_16_ = 0.09, *p* = 0.77, respectively) we did observe some interactions. For adult contact behavior, there was a trend such that infants with a greater change in locomotor behavior between conditions were observed in contact with their mate more often than infants with smaller changes in locomotor behavior, unless the infant experienced adversity during development [standardized β = −1.30, CI 95% = (−1.30, −1.29), *p* = 0.060] (Table 6). We ran the same model for adult proximity and found a stronger relationship between infant locomotor behavior and adult behavior [standardized β = −0.777, CI 95% = (−0.779, −0.776), *p* = 0.045] (Figure 7). To confirm the effect was related to infants’ primary attachment figure, we also ran the model comparing locomotor behavior when mom was present to the empty condition and found no trends for either contact [standardized β = −0.13, CI 95% = (−0.14, −0.13), *p* = 0.85] or proximity [standardized β = −0.496, CI 95% = (−0.499, −0.493), *p* = 0.48].

**TABLE 6:** Summary of results for Experiment 2.

Predictor	Outcome	β (standardized)	SE	*p*
	*Contact with mate*			
Adverse early experience		0.06	0.04	0.19
Change in IOF locomotor behavior (father)		0.59	>0.001	0.10
Change in IOF locomotor behavior (mother)		>0.001	>0.001	0.97
Change in IOF locomotor behavior (father) ^∗^ adverse early experience		–1.30	0.001	0.060^∗^
Change in IOF locomotor behavior (mother) ^∗^ adverse early experience		–0.18	0.001	0.79
	*Proximity to mate*			
Adverse early experience		0.42	0.04	0.12
Change in IOF locomotor behavior (father)		0.66	0.001	0.061^∗^
Change in IOF locomotor behavior (mother)		0.45	0.001	0.52
**Change in IOF locomotor behavior (father) ^∗^ adverse early experience**		**−0.78**	**0.001**	**0.045**
Change in IOF locomotor behavior (mother) ^∗^ adverse early experience		–0.50	0.001	0.48

*Linear mixed models (*N* = 25) of adult affiliative behavior from infant behavior. Boldface indicates statistically significant predictors and ^∗^ indicates a trend. Adverse early experience was coded as 1 and typical experience was coded as 0.*

## Discussion

### Experiment 1

The current study examined attachment behavior in coppery titi monkeys as a potential animal model of human attachment. We tested whether infant behavior during an IOF test, modified to resemble the Ainsworth Strange Situation Paradigm, could be used to explain variability in adult participation in a novelty response task depending on whether their pair mate was present or absent. Our results should be interpreted as exploratory until more data can be collected, and we can test our hypothesized models. In concordance with human literature reporting stability in attachment behavior from parent–infant bonds to adult partner bonds, we found support for our hypothesis that highly reactive infant titi monkeys are also highly reactive as adults within the current dataset (Waters and Merrick, 2000; Fraley, 2002). These subjects exhibited a dramatic change in locomotor, grate touch, grate zone, and vocalization behavior between the alone and father condition in the IOF and a dramatic change in participation rates between the alone and partner conditions during the novelty response task. Our results suggest that the type of attachment behavior which an infant titi monkey displays with their father is indicative of the type of attachment behavior they will share with their adult pair mate. Additional data are needed to test our models and confirm this relationship as predictive rather than correlational.

Although infant vocal behavior, location in the field, and grate touch behavior were not able to explain variability in adult behavior, we found an interesting relationship between infant locomotor behavior in the IOF and adult response to novelty. Considering locomotion as an accepted measure for anxiety-like behavior and the consistency of this behavior with participation in the novelty response task we believe this result is in line with traditional Attachment Theory (Barros and Tomaz, 2002). In the IOF paradigm, nearly all infants locomoted less when they had visual access to their father than when they did not, but the amount that infants locomoted during the father condition varied by individual. Infants with the lowest levels of locomotion in the father condition were the same adults that participated the most in the novelty response task when their pair mate was present. There was no apparent relationship between grate zone behavior when the father was present and adult participation; therefore, we believe this decrease in motor activity was not related to the infant’s desire to be in proximity to their father, but rather a more generalized decrease in anxiety-like behavior. Along with Bowlby’s initial theory, many studies have pointed out consistencies between infant attachment style and trait outcomes such as anxiety, depression, and the big five personality traits (Hagekull and Bohlin, 2003; Picardi et al., 2005). Although social condition alone was unable to explain task participation, there was an interaction showing that the relationship between infant behavior and adult performance was stronger when the attachment figure was present than when the subject was alone. This suggests that some of our subjects are generally less reactive than others and their ability to cope with strange or novel situations is related to the kind of relationship they have with their attachment figures.

Our results also illustrated a relationship between some infant behaviors and adult participatory behavior when separated from their attachment behavior. When we analyzed infant behavior in the presence of the infant’s mother, we found relationships between grate zone behavior and adult participation, as well as an interaction between grate touching behavior and adult participation. We believe these effects are likely due to temperament rather than attachment behavior because we did not observe the same effects for the father condition.

Experiment 1 also explored the relationship between observed affiliation between the subjects and their current pair mate, distress following involuntary separation from their pair mate, and participation in the novelty response task. Although our task elicited the expected anxiety-like response observed in previous studies (Arias del Razo et al., 2019), we were unable to confirm our hypothesis that individuals in pairs exhibiting higher rates of affiliative behavior would be more distressed during a partner’s absence, and consequently less likely to participate in the novelty response task. However, our results did show an interesting relationship between affiliative behavior and adult anxiety-like behavior regardless of whether or not their pair mate was present during the task. More affiliative subjects in this dataset were less likely to participate in the task than their less affiliative counterparts. Interestingly, this effect was the opposite for pairs in their second or third pairing but given the small sample size of the current study, the relationship between pair experience, affiliation, and anxiety-like behavior should be further explored. It is also difficult to know if this effect was driven by pair experience or if there is an effect of age on anxiety-like behavior in titi monkeys, given that the subjects with more pair experience were also the oldest in the study. Studies in humans suggest that older individuals report fewer symptoms of generalized anxiety (Byers et al., 2010; Miloyan et al., 2014).

The role of social buffering during an anxiety response is complex. Ditzen et al. (2008) investigated differential psychological and physiological responses to the Trier Social Stress Test in individuals with anxious or avoidant attachment styles according to Attachment Theory. Their findings suggested some interactions between attachment behavior and stress response, but they were inconclusive in terms of whether or not these individuals were specifically responding to the social support differently. Similarly, in our study, we were unable to see a clear change in behavior as a result of the presence or absence of their pair mate, but we did see an interaction between attachment behavior and test condition such that the individuals most calmed by their fathers also tended to receive the most benefit from having their pair mate present. Although the effect of social condition did not stand out in our experiment, we do not believe this to be indicative of a lack of social support from their pair mate.

Adult titi monkeys are more likely to engage with a novel object and exhibit lower levels of autonomic arousal when their pair mate is present (Cubicciotti and Mason, 1975; Fragaszy and Mason, 1978; Hennessy et al., 1995). It is possible that our testing paradigm did not initiate a strong enough reaction to involuntary separation to inhibit behavioral response during testing. However, we believe it is more likely that the lack of statistical evidence of social support in the current study is due the specific individuals in the subject pool. Of our 11 subjects, only 7 of them were engaged in their first adult pair bond while the other 4 were currently paired with their second or even third pair mate. Prior to the design of this experiment, we did not expect titi monkey attachment behavior to change over the course of multiple pair bonds. New data from our lab show a clear increase in affiliative behaviors for males in their second pair bond compared to their first (Witczak et al., in preparation). These new findings indicate the need for further investigation of the flexibility of attachment behavior in adult titi monkeys.

### Experiment 2

In Experiment 2, we tested whether infant attachment behavior was directly related to adult affiliative behavior. To our knowledge, this is one of the first studies indicating a change in attachment behavior, as described in Attachment Theory, from infancy to adulthood resulting from adverse life events in NHPs. As we observed in Experiment 1, our results indicated that, of all the infant behaviors we measured, only locomotive behavior was indicative of adult behavior. Until we are able to test this hypothesis on a new data set, we can only interpret these results as they relate to these specific animals, not the entire population. For these titi monkeys, infant locomotion, or anxiety-like behavior, trends with adult proximity behavior during the first 6 months of their first pair bond. There appears to be a negative correlation between the extent to which the infant is “calmed” by their father’s presence and their adult proximity seeking behavior. Perhaps more interesting is the significant interaction between this trend and adverse early experience in titi monkeys. For subjects with typical early experience, the less they locomoted when their father was present compared to his absence (i.e., how “calmed” they were by their attachment figure), the more affiliative they were in pairs as adults. However, infants with adverse early experience, but similar locomotor responses during the IOF test, did not follow this pattern. While affiliative behaviors did not differ by group (adverse vs. typical), the developmental trajectory appears to be altered. Our sample size was not sufficient to thoroughly assess whether the interaction was driven by some infants responding to adverse experience by becoming more affiliative or less affiliative, but we can see that their infant attachment behavior is incongruent with their adult attachment behavior. Given that the current study did not control for genetic variability between our adverse and typical groups, we are unable to conclude if the adverse experiences themselves attributed to incongruent attachment behavior or if group differences were due to genetic differences (Barbaro et al., 2017).

Human and NHP research have documented changes in attachment behavior resulting from adverse early experiences or negative life events (Harlow, 1964; Bowlby, 1982; Weinfield et al., 2000). Adverse early experiences related to the caregiver have been shown to alter specific brain regions related to social behavior (Yan et al., 2017). Macaque infants that experienced abusive behavior from their mothers illustrated higher rates of anxiety-like and proximity seeking behavior throughout development (McCormack et al., 2006). It is difficult to tell if a similar effect was occurring in our subjects, but there is evidence in NHPs that adverse experiences during critical developmental periods can have long-term implications for the HPA axis and stress-related behavior (Sanchez et al., 2010; Koch et al., 2014). Unfortunately, none of these studies followed their subjects through development into adulthood to investigate possible effects on social behavior so we do not currently have any insight as to how adverse early experience is affecting titi monkey neurobiology. We believe our findings suggest that titi monkeys could play an integral role in understanding these neurobiological changes specifically related to pair bonding.

### Limitations

Despite the benefits of studying titi monkeys in a laboratory setting, the current study had several limitations. Most importantly, investigating attachment behavior can be difficult with a small sample size because of the natural variation in behavior. All of our reported results are exploratory and should be considered as hypothesis-generating rather than confirmatory. In humans, insecure attachments are observationally and biologically very different from secure attachments. For example, infants with some types of attachment insecurity exhibit dramatic increases in proximity seeking behavior while others exhibit decreases. A comprehensive analysis of adult attachment styles in non-clinical European subjects classified 58% of the population as secure and divided the other 42% of subjects into four categories of insecure attachments (Bakermans-Kranenburg and van IJzendoorn, 2009). With a sample size of 25 we were unable to confidently classify our subjects into different categories of attachment and as such we were not able to control for behaviors linked to those individual differences. More research is needed in order to fully understand the variability of attachment behavior in titi monkeys.

## Conclusion

Although a lot of work has been done in rodents and other NHP models investigating the effects of adverse early experiences on social behavior and health outcomes there is still a great need for direct investigation of the development of adult pair bonds (Rincón-Cortés and Sullivan, 2016; Fraley, 2019; Hennessy et al., 2019). Our current findings lay the groundwork for a NHP model of the attachment system across the life span. As noted in the section “Limitations,” our sample size was too small to test our models’ predictive ability and future studies will need to address this in order to understand the relationship between infant and adult attachment behavior. We ran power analyses for both Experiment 1 and Experiment 2 and found that future studies would need samples sizes of 50 and 22, respectively, for statistical power of 0.80. Future studies in monogamous NHPs have the potential to precisely identify key periods for the development of the attachment system in a way that has proven very difficult in humans. Identifying these periods will expand our knowledge of how social attachments affect our biology and provide more opportunities to test potential interventions.

## Data Availability Statement

The datasets generated for this study are available on request to the corresponding author.

## Ethics Statement

The animal study was reviewed and approved by the University of California, Davis Institutional Animal Care and Use Committee.

## Author Contributions

LS collected the data, designed and performed the analyses, and wrote the first draft of the manuscript. KB obtained the funding for the project, participated in the research and analytical design, and edited the manuscript.

## Conflict of Interest

The authors declare that the research was conducted in the absence of any commercial or financial relationships that could be construed as a potential conflict of interest.

## Footnotes

1 www.behaviortracker.com

## Supplementary Material

TABLE S1 Results from all log-likelihood tests for model comparison.
