# Supplementary material for: An Animal Model for Mammalian Attachment: Infant Titi Monkey (Plecturocebus cupreus) Attachment Behavior Is Associated With Their Social Behavior as Adults
Source: Front Psychol. 2020 Jan 28;11:25. doi: 10.3389/fpsyg.2020.00025 (PMC6997438; doi:10.3389/fpsyg.2020.00025)
Supplement: TABLE S1 — Results from all log-likelihood tests for model comparison. [file Table_1.docx]

|  | AIC | Log-likelihood | DF | Chi-squared | P-value |
| --- | --- | --- | --- | --- | --- |
| 1A – All Trials |  |  |  |  |  |
| Participation ~ *1+ (1\|Subject)* | 181.65 | -87.823 | 3 |  |  |
| Participation ~ *Condition*Order*Pair Experience*Sex *Contact*Proximity + (1\|subject)* | 180.85 | -76.427 | 14 | 22.793 | 0.69 |
| Participation ~ *Condition*Order*Pair Experience *Contact *Proximity + (1\|subject)* | 177.59 | -76.796 | 12 | 10.63 | **0.005** |
| Participation *~ Condition*Pair experience*Contact *Proximity + (1\|subject)* | 184.22 | -82.11 | 10 | 11.42 | 0.12 |
| 1B – Easy Trials |  |  |  |  |  |
| Participation ~ *1+ (1\|Subject)* | 154.72 | -74.36 | 3 |  |  |
| Participation ~ *Condition*Order*Pair Experience*Sex *Contact*Proximity + (1\|subject)* | 168.96 | -70.48 | 14 | 0.68 | 0.71 |
| Participation ~ *Condition*Order*Pair Experience *Contact *Proximity + (1\|subject)* | 165.64 | -70.82 | 12 | 0.51 | 0.78 |
| Participation *~ Condition*Pair experience*Contact *Proximity + (1\|subject)* | 162.15 | -71.08 | 10 | 2.17 | 0.34 |
| Participation *~ Condition*Pair experience*Contact*  *+ (1\|subject)* | 160.32 | -72.16 | 8 | 0.68 | 0.71 |
| 1C – Difficult Trials |  |  |  |  |  |
| Participation ~ *1+ (1\|Subject)* | 211.67 | -102.84 | 3 |  |  |
| Participation ~ *Condition*Order*Pair Experience*Sex *Contact*Proximity + (1\|subject)* | 209.08 | -90.54 | 14 | 0.77 | 0.68 |
| Participation ~ *Condition*Order*Pair Experience *Contact *Proximity + (1\|subject)* | 205.84 | -90.92 | 12 | 3.07 | 0.22 |
| Participation *~ Condition*Pair experience*Contact *Proximity + (1\|subject)* | 204.91 | -92.46 | 10 | 10.12 | **0.006** |
| Participation *~ Condition*Pair experience*Contact*  *+ (1\|subject)* | 211.02 | -97.51 | 8 | 10.65 | 0.06* |
| 1D – Infant Behavior with Dad |  |  |  |  |  |
| Participation ~ *1+ (1\|Subject)* | 211.67 | -102.84 | 3 |  |  |
| Participation ~ *Condition*Grate Touch*Grate Zone *Locomotion*Vocalizations*Order*Sex + (1\|subject)* | 192.01 | -80.01 | 16 | 1.04 | 0.59 |
| Participation ~ *Condition*Grate Touch*Grate Zone *Locomotion*Vocalizations*Order + (1\|subject)* | 189.05 | -80.5 | 14 | 0.88 | 0.64 |
| Participation ~ *Condition*Grate Touch*Grate Zone *Locomotion*Vocalizations + (1\|subject)* | 185.93 | -80.97 | 12 | 22.55 | **> 0.001** |
| Participation ~ *Condition*Grate Touch*Grate Zone *Locomotion + (1\|subject)* | 204.48 | -92.24 | 10 | 12.24 | **0.003** |
| 1E – Infant Behavior with Mom |  |  |  |  |  |
| Participation ~ *1+ (1\|Subject)* | 211.67 | -102.84 | 3 |  |  |
| Participation ~ *Condition*Grate Touch*Grate Zone *Locomotion *Order*Sex + (1\|subject)* | 208.22 | -91.92 | 14 | 0.3 | 0.86 |
| Participation ~ *Condition*Grate Touch*Grate Zone *Locomotion *Order + (1\|subject)* | 205.23 | -92.07 | 12 | 0.34 | 0.84 |
| Participation ~ *Condition*Grate Touch*Grate Zone *Locomotion + (1\|subject)* | 202.52 | -92.24 | 12 | 21.19 | **0.002** |
